# Diaphragmatic Palsy

**DOI:** 10.3390/diseases6010016

**Published:** 2018-02-13

**Authors:** Laxmi Kokatnur, Mohan Rudrappa

**Affiliations:** 1 Department of Neurology, Louisiana State University Health Science Center, 1501 Kings Highway, Shreveport, LA 711031, USA; 2 Department of Neurology, Overton Brooks VA Medical Center, 501 E Stoner Ave, Shreveport, LA 71101, USA; 3 Department of Neurology, Mercy Hospital, 100 Mercy Way, Joplin, MO 64804, USA; 4 Department of Pulmonary and Critical Care Medicine, Louisiana State University Health Science Center, 1501 Kings Highway, Shreveport, LA 711031, USA; 5 Department of Pulmonary and Critical Care Medicine, Overton Brooks VA Medical Center, 501 E Stoner Ave, Shreveport, LA 71101, USA; 6 Department of Pulmonary and Critical Care Medicine, Mercy Hospital, 100 Mercy Way, Joplin, MO 64804, USA

**Keywords:** diaphragmatic palsy, phrenic nerve injury, respiratory failure, pulmonary function tests

## Abstract

The diaphragm is the primary muscle of respiration, and its weakness can lead to respiratory failure. Diaphragmatic palsy can be caused by various causes. Injury to the phrenic nerve during thoracic surgeries is the most common cause for diaphragmatic palsy. Depending on the cause, the symptoms of diaphragmatic palsies vary from completely asymptomatic to disabling dyspnea requiring mechanical ventilation. On pulmonary function tests, there will be a decrease in the maximum respiratory muscle power. Spirometry shows reduced lung functions and a significant drop of lung function in supine position is typical of diaphragmatic palsy. Diaphragmatic movements with respiration can be directly visualized by fluoroscopic examination. Currently, this test is being replaced by bedside thoracic ultrasound examination, looking at the diaphragmic excursion with deep breathing or sniffing. This test is found to be equally efficient, and without risks of ionizing radiation of fluoroscope. Treatment of diaphragmatic palsy depends on the cause. Surgical approach of repair of diaphragm or nonsurgical approach of noninvasive ventilation has been tried with good success. Overall prognosis of diaphragmatic palsy is good, except when it is related to neuromuscular degeneration conditions.

## 1. Introduction

The diaphragm is a dome-shaped musculo-fibrous structure located between the thoracic and abdominal cavity. Being the primary muscle of respiration in mammals, it is vital organ for their survival. The word “diaphragm” is derived from the Greek words “dia”, meaning in between, and “phragma”, meaning fence. Diaphragm constitutes the floor of the thorax and the roof of the abdomen. Diaphragmatic palsy is the loss of its muscular power. This can arise either from weakness of the diaphragmatic muscle fibers itself or injury to its sole nerve supply, phrenic nerve. Depending on the severity of the paralysis, and whether the weakness is either unilateral or bilateral, patients can have varied clinical manifestations from completely asymptomatic noticed incidentally on routine chest X-ray to permanent respiratory failure requiring permanent ventilator assistance. In this review, we discuss the causes of diaphragmatic palsy along with evaluation and management of patients with diaphragmatic palsy (DP).

## 2. Anatomy

Although a clear anatomical distinction is not evident, the diaphragm functions as two separate units (right and left) and has its own separate vascular supply and innervation. The diaphragm has two distinct parts, central tendinous and peripheral muscular. The muscular part has three distant muscle groups divided into sternal, costal and lumbar. The anterior sternal part consists of two tiny muscle bundles derived from the back of xiphoid process. The lateral costal part originates from the internal surface and superior margins of the six inferior ribs [1]. On the posterior side, the lumbar portion is derived from medial, intemedial and lateral diaphragmatic pillars. The central tendinous part is a C-shaped aponeurotic structure with three leaflets. Unlike other tendons in the body, this tendinous part merge with the surrounding muscle fiber of diaphragm without any bony connection. The ventral attachment of the diaphragm is located at a more cranial location compared to its dorsal attachments giving a sloping profile on sagittal section. On the cranial side, the diaphragm merges with the pulmonary pleura, and on the caudal side it merges with the peritoneum. The diaphragm has several openings enabling structures to pass from the thorax to the abdomen.

There are three main opening in diaphragm allowing the communication of thoracic cavity and abdominal cavity organs. The largest opening is present on the right hemidiaphragm at the level of eight thoracic vertebrae through which the inferior vena cava the enters the thorax from the abdomen, and joins the right atrium. In the posterior midline location between the two crus of diaphragm at the level of tenth thoracic, there is the aortic opening through which the descending thoracic aorta enters the abdomen from the thorax. This opening also contains the thoracic duct and azygous vein which enters the thoracic cavity from the abdomen. Between the fibers of the right crus of the diaphragm, there is the esophageal hiatus through which the esophagus enters the abdominal cavity to join stomach.

The main vascular supply for the diaphragm comes from the bilateral phrenic arteries arising directly from the descending thoracic aorta. The diaphragm is also supplied by tributaries of internal mammary and pericardiophrenic arteries. Venous drainage is through the phrenic veins which ultimately drain into the inferior vena cava.

During gestation, the diaphragm starts developing during the seventh week and is complete by the tenth week. It is derived from three structures the septum transverse, right and left pleuroperitoneal membrane and the dorsal mesentery of the esophagus. The diaphragmatic muscles arise from the third to fifth cervical myotomes and hence supplied by third to fourth cervical roots through the phrenic nerve [2].

## 3. Functions

From the functional perspective, the diaphragm has two distinct regions, the crural part which is primarily involved in respiration, and the costal part which is primarily involved in preventing Gastroesophageal reflex [1]. During inspiration, the diaphragmatic muscle contracts leading to both caudal displacement of the muscle and alternation of curvature of muscle leading to flattened and thickened muscle. The resulting expansion of the thoracic cavity will lead to further drop in negative intrathoracic pressure. This positive pressure gradient from normal atmospheric pressure to negative intrathoracic pressure will lead to movement of air into lungs leading to inspiration. At the end of inspiration, the diaphragmatic muscle relaxes and passive constriction of the thoracic cavity due to elastic recoil will increase the intrathoracic pressure will lead to egresses of air from the respiratory system causing expiration. Under normal conditions, the diaphragm acts like a piston within the chest, generating flow as its dome descends within the thoracic cavity, while it displaces the abdominal contents caudally and elevates the lower thorax. The muscular component of the diaphragm has equal proportions of slow, fatigue-resistant (Type I) and fast (Type II) fibers enabling the muscle to in both low-intensity perpetual cycles of breathing and, in more rapid and strenuous power generation such as talking, singing, sneezing, defecation and in situations of acutely-increased ventilation. Although the diaphragm is generally under involuntary neural control from the central nervous system, it can be augmented by additional voluntary input. Voluntary contraction of the diaphragm also increase the intraabdominal pressure and is required for other vital functions like vomiting, urination, and defecation.

## 4. Nerve Supply

The phrenic nerve is the sole nervous supply to the diaphragm and acts as both a sensory and motor nerve. As diaphragmatic muscles take origin from cervical myotomes, its nerve supply is also derived from cervical roots [3]. Typically the cervical roots of C3 to C5 give rise to the phrenic nerve, but can have contributions from C2 to C7 levels. The phrenic neurons are housed in lamina IX of the ventral horn in the cervical spinal cord, and receive information via presynaptic contacts in the medulla. After originating in the neck, the phrenic nerve descends down anterior to scalene muscle and enters the thorax between the subclavian artery and vein. In the mediastinum, the right phrenic nerve runs anterior to the brachiocephalic trunk located lateral to the right atrium, and then runs through the caval opening of diaphragm enter the abdominal cavity. The left phrenic nerve runs caudally along the left ventricle and pierces the diaphragm lateral to heart border. From the abdominal side of the diaphragm, the phrenic nerves divide into four major trunks: anterolateral, posterolateral, sternal, and crural trunk [3,4]. 

## 5. Diaphragmatic Palsy (DP)

Although external intercostal muscles aid in inspiration, the diaphragm is the primary muscle of respiration and its weakness can impede the vital respiratory functions. When the diaphragm is weak, the caudal displacement during inspiration is impaired or absent. This limits the expansion of thoracic cavity and result is suboptimal inspiration resulting in hypoventilation. As the diaphragm functions as separate units, right and left hemidiaphragm, paralysis on both sides of the hemidiaphragm will cause significant respiratory failure, but paresis of one hemidiaphragm can be completely asymptomatic. The hemi-diaphragmatic weakness is compensated by opposite hemi-diaphragmatic functions and recruitment from external intercostal muscles. Diaphragmatic palsy can be a result of either intrinsic diaphragmatic muscle fibers weakness, or damage to the phrenic nerves. Unilateral weakness of one of the hemidiaphragms is more common than bilateral weakness. Depending on the cause, the DP can be either temporary or permanent. The causes of diaphragmatic weakness can be divided into the following categories:

∙ Traumatic

**Figure 1:**
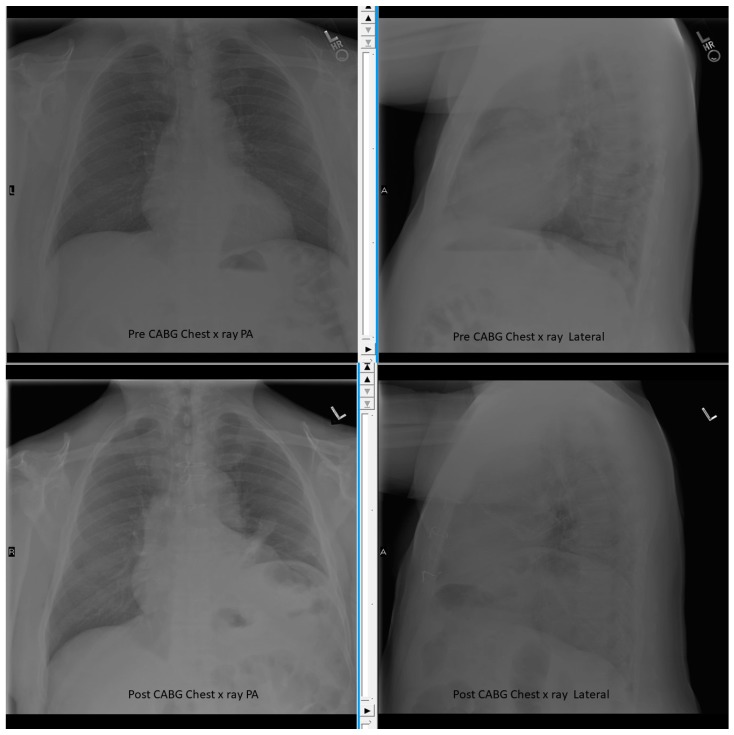
Chest X-ray Pre and Post Cardiac Bypass Surgery (CABG) showing elevated left diaphragm after surgery. The sternal metal suture wires also seen.

Traumatic injury to the phrenic nerve either during thoracic surgery or mechanical trauma is the most common cause of diaphragmatic weakness. Traumatic causes are usually unilateral. They are often asymptomatic only to be discovered during routine follow up chest X-ray after surgery (Figure 1). Phrenic nerve palsy is very common after cardiac bypass surgery is the most common of all traumatic causes. As the phrenic nerve is located very close to the heart, cold cardioplegia used during cardiac bypass will cause phrenic nerve cold injury called phrenic nerve frostbite and lead to impaired conduction on impulses. Due to the long course of the left phrenic nerve in the thorax, left-side diaphragmatic weakness is more common compared to right side [5,6,7,8,9,10]. Any mediastinal procedures, esophageal surgeries or lung transplantation carry the risk of phrenic nerve injury and diaphragmatic weakness [11,12,13,14,15,16,17,18]. Penetrating trauma or gunshot injuries to the thorax can also result direct diaphragmatic muscle rupture leading to DP or in rare occasions can cause phrenic nerve damage leading to DP. 

∙ Compression

**Figure 2:**
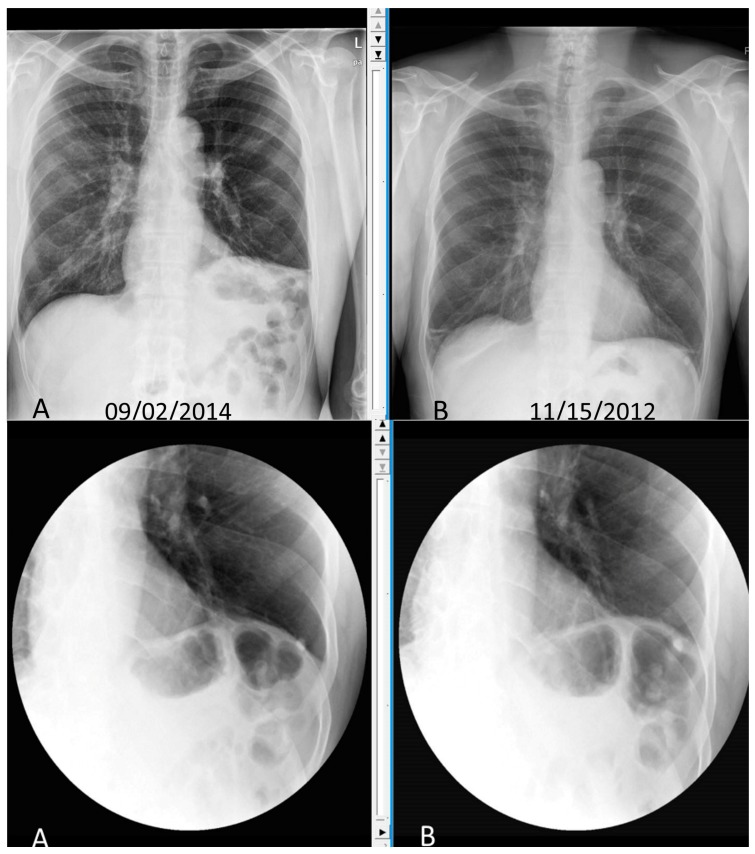
Chest X-ray and Static Sniff test of patient with cervical spondylosis leading to phrenic nerve compression and left diaphragmatic palsy. **Top panel** showing chest X-ray with elevated left diaphragm (**A**) compared to normal position on chest X-ray done 2 years back (**B**). **Lower panel**. Static Sniff test images in expiration (**A**) and after sniffing (**B**) showing no movement of diaphragm.

Any space-occupying lesion in the thorax (both mediastinal or lung mass) in close proximity to the phrenic nerve can cause phrenic nerve damage by either direct infiltration or extrinsic compression. Phrenic nerve involvement is seen in 5% of lung cancer patients [19,20]. Diaphragmatic palsy is also described in patients with cervical spondylosis resulting in compressive neuropathy (Figure 2) [21]. Other causes of compressive damage to phrenic nerve include aortic aneurysm, substernal goiter and von Recklinghausen disease [19].

∙ Neurological

The phrenic neurons can be involved in demyelinating diseases like multiple sclerosis [22]. Any type of cervical cord injury can also damage the phrenic neurons. The severity of injury depends on the level of injury. While half of patients with injury above the level of C3/C4 require mechanical ventilation, only 15% of patients with injury below the level of C5 require any respiratory assistance [23]. Other neurological disease affecting lower motor neurons also can cause bilateral phrenic nerve palsy leading to respiratory failure and requiring ventilator support. One third of patients with Gillian–Barré syndrome have respiratory failure, and 5% of patients with amyotrophic lateral sclerosis have respiratory failure [23,24]. Disease of the neuromuscular junction like myasthenia gravis also can cause diaphragmic palsy. Other rare causes in this neurological category are syringomyelia, paraneoplastic motor neuropathies and hereditary myopathies and spinal muscular atrophies. 

∙ Infectious

Poliomyelitis was one of the major causes of permanent respiratory failure in the pre-vaccination era. With widespread adaptation of vaccinations against poliomyelitis in children, this is rarely seen in present era. Other arbovirus like West Nile virus and dengue virus can cause phrenic nerve palsy and incidence of these infections are a rising trend in the United States recently [25]. Lyme disease is are reported to cause bilateral phrenic nerve palsy [26]. 

∙ Inflammatory

Many systemic conditions can lead to DP. Sarcoidosis, collagen vascular disease, dermatomyositis either involve the phrenic nerve causing neuropathy or can directly cause diaphragmatic myopathy leading to respiratory failure [27]. Shrinking lung syndrome, a rare complication of systemic lupus erythematosus, is also described in other connective disease like Sjogens syndrome, scleroderma and rheumatoid arthritis. Shrinking lung syndrome is characterized by progressive dyspnea, pleurisy and elevated diaphragm without any parenchymal abnormality but shows evidence of diaphragmatic weakens [28]. 

∙ Idiopathic

In nearly 20% of cases, no obvious cause for diaphragmatic palsy can be identified despite extensive despite extensive investigations and are referred as “idiopathic”. Hypothyroidism and malnutrition are reported to cause DP with complete recovery with appropriate treatment [29,30]. 

∙ Critical Illness Related Neuromyopathy

Critically ill patients intensive care unit show evidence of DP. Critical illness related Neuro-myopathy leading to DP can cause failure to wean from mechanical ventilation, increase the length of stay in the intensive care unit, increase the length of stay on the ventilator. The overall mortality and morbidity is these subsets of patients is also reported to be high [31,32,33,34,35]. Diaphragmic muscle atrophy is documented within 24 h of mechanical ventilation in both human and animal studies. Various mechanisms like oxidative stress, decreased protein synthesis and exaggerated proteolytic pathway have been described for this muscle atrophy [36,37,38,39,40]. It is also postulated that some of these critically sick patients might have preexisting diaphragmatic weakness that gets pronounced with added inflammatory response during illness. 

## 6. Clinical Features

Symptoms of diaphragmatic weakness vary depending on the cause and duration of the disease, and whether one or both hemidiaphragms are affected. Most patients with unilateral hemi-diaphragmatic weakness are asymptomatic. They are usually detected incidentally on a routine chest X-ray showing elevated hemidiaphragm. However patients with compromised cardiopulmonary reserve due to coexisting disease can report exertional breathlessness, orthopnea or sleep disturbances. On the contrary, most patients with bilateral diagrammatic weakness report dyspnea of varying severity [41,42,43,44]. The dyspnea is associated with debilitating orthopnea. In patients with DP on lying supine, there is extrinsic compression of thoracic cavity by the abdominal organs due to loss of caudal gravitational pull. Also, on lying supine, the chest wall will be in close physical contact with the floor will further preventing thoracic excursion. This positional worsening of respiratory functions will lead to choking sensation on lying down flat on their back. Some patient experience severe breathlessness on immersion in water filled up to waist level [45]. Most patients with bilateral DP sleep in recliner. They also report fatigue, hypersomnia and depression due to sleep disturbance. Due to limited thoracic excursion, patients with diaphragmatic palsy are also prone to frequent lung infections. Chronic hypoventilation can cause hypoxia and hypercapnia and occurs more during sleep. It this persists for a long time, the patients can show clinical features of right heart failure. Clinical examination is usually not revealing except in severe bilateral diaphragmatic weakness wherein paradoxical respiratory movements can be seen. In supine position during inspiration, the abdomen moves inward instead of outwards due to due to diaphragm getting sucked into the expanding thoracic cavity [23]. 

## 7. Investigation

**Figure 3:**
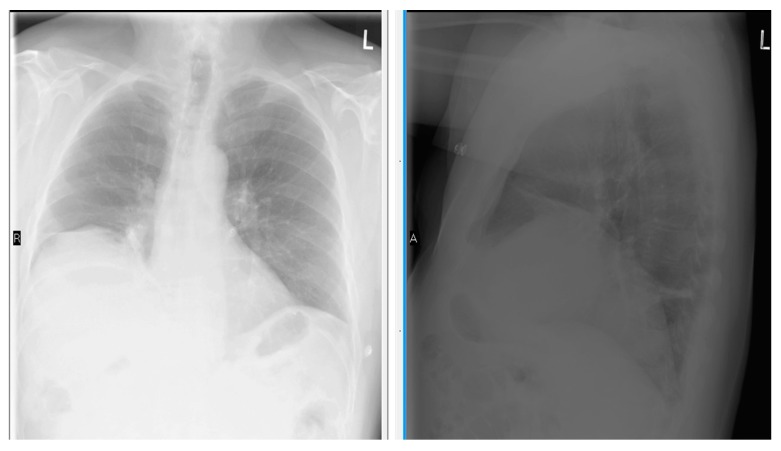
Chest X-ray Posterior-Anterior and Lateral view showing right diaphragm located more than 2 intercostal spaces compared to left side.

**Chest radiograms**. Around 90% of unilateral diaphragm palsy is diagnosed based on an elevated hemidiaphragm on routine chest X-rays [46]. In normal individuals, diaphragm position obliquely with dome of the dome of the right diaphragm is at the level of the fifth rib anteriorly, and the level of the tenth rib posteriorly. The left hemidiaphragm is usually located one intercostal space lower than the right hemidiaphragm. If one hemidiaphragm is weak, then the normal negative intrathoracic pressure will suck the diaphragm cranially into the thoracic cavity. So, the paralyzed diaphragm will be at a higher level. If the right side is paralyzed, the distance between the right and left diaphragm will be more than two intercostal spaces, and if the left side is paralyzed both hemidiaphragms will appear on the same level (Figure 3). In bilateral weakness, both hemidiaphragms will at a higher level than expected and might be missed on a static chest X-ray. Sometimes a deep costophrenic and craniovertebral angle can be noted due to increased curvature of diaphragm due to its cranial displacement. DP can also cause secondary atelectasis of the lower sub-segments [46].

**Fluoroscopic test**: On tidal breathing in normal individuals, diaphragmatic contraction will lead to the caudal descent of both hemidiaphragms by at least one intercostal space. On deep breathing or sniffing, the descent is more pronounced, and is more rapid. A paralyzed hemidiaphragm will not show any movements during sniff examination, or can show paradoxical movements in opposite directions (video1 supplementary file). Fluoroscopic sniff test requires significant patient effort and cooperation. Patients with complete bilateral DP have orthopnea and cannot tolerate the supine position needed for this procedure. Also, due to risks of ionizing radiation on health, this test is being replaced by an ultrasound examination of the diaphragm. 

**Ultrasound of thorax:** The use of ultrasound technology for evaluation of thoracic pathology has been increasing in the recent time. Ultrasound of diaphragm is noninvasive, simple and can be portable. This has replaced the fluoroscopic sniff test as the test of choice especially in young patients due to concerns of cancer due to risk of ionizing radiation by fluoroscopic examination. The diaphragm appears as a thick echogenic line in M mode ultrasound examination [47]. Diaphragm thickness varies with inspiratory effort and level of contraction and at total lung capacity it is around 1.7 to 2.2 cms. Any thickness of less than 15 mm suggests diaphragmatic weakness. During inspiration, diaphragmatic muscle contracts and its thickness increase by at least 20% compared to its thickness at the end of quiet expiration. This dynamic change in its thickness with respiration can be measured by using M mode and expressed as percentage change of thickness with deep breathing. Less than 20% increase in diaphragmatic thickness with inspiration suggests diaphragmatic weakness. As the diaphragm descends to the abdominal cavity with inspiration, reduction in its descent also suggest its weakness (video 2 Supplementary files). Diaphragmatic excursion of less than 3.7 cms in females and less than 4.7 cms in males suggest diaphragmatic weakness [47,48,49,50,51,52]. In severe weakness, paradoxical movements of the diaphragm can be visualized easily, and can be measured in M mode. 

**Figure 4:**
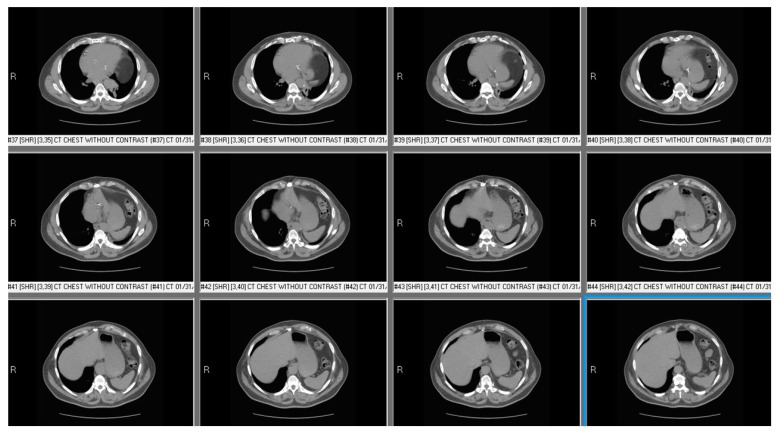
Computed Tomography (CT) chest of patient with left diaphragmatic palsy showing abdominal contents beside heart and at higher level compared to liver suggesting left diaphragmatic palsy.

**Computed Tomography (CT) of Chest**: CT of the chest should be done in all patients with diaphragm weakness to rule out any thoracic pathology causing compressive neuropathy of phrenic nerve. Unfortunately with the present CT technology, the diaphragm cannot be directly visualized in static CT scans (Figure 4). With inspiratory and expiratory cuts, the displacement of diaphragmatic structures can be noted and may suggest DP.

**Figure 5:**
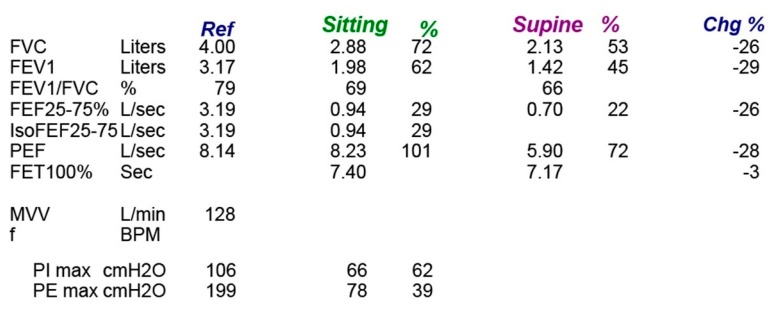
Spirometry of patient with bilateral diaphragmatic palsy showing significant drop of lung functions on supine position.

**Pulmonary function tests**: As the diaphragm accounts for 80% of the muscular power of respiration, pulmonary functions tests can easily detect DP. For all patients with suspected diaphragmatic palsy, spirometry should be done in both sitting and supine position. In unilateral hemidiaphragm, forced vital capacity will be reduced by 30% predicted, and in bilateral paralysis it further decreases by 75% predicted. On supine position in normal individuals, the lung function declines by less than 15% due to the restriction of thoracic expansion and external pressure from abdominal structures. In patients with diaphragmatic palsy, forced vital capacity will further decrease by 15% to 20% in unilateral weakness, and 20% to 30% in bilateral weakness (Figure 5). This significant reduction of vital capacity in supine position also correlates with tans-diaphragmatic pressure. In patients with DP, total lung capacity will be reduced to 70% to 80% of that predicted in unilateral weakness and 30% to 50% of that predicted in bilateral weakness. Residual volume and functional residual capacity are usually normal in unilateral DP but can be bilateral cases [53,54,55].

Maximum (static) inspiratory pressure. Maximum Inspiratory pressure is the pressure recorded at the mouth during a maximal inspiratory effort against a closed mouthpiece. Due to its simplicity, it is widely used as a test of respiratory muscle function. It represents the combined effort of all inspiratory muscles rather than isolated diaphragmatic contraction. Maximum inspiratory pressure of more than 80 cms of water excludes any significant diaphragmatic weakness. In unilateral weakness, this pressure will be reduced to about 60% of predicted and in bilateral cases it will be reduced to 30% predicted. Maximal expiratory pressure also can be reduced in isolated diaphragmatic palsy due to the suboptimal tension properties of the expiratory muscles at the reduced lung volumes rather than direct myopathic process [56,57,58]. 

Twitch diaphragmatic pressure. This is the negative pressure generated by diaphragmatic contraction in response to phrenic nerve stimulation. Phrenic nerve is electrically stimulated and simultaneous esophageal pressure and gastric pressure are recorded. The difference between these pressures gives the twitch trans diaphragmatic pressure and directly correlated with DP. Despite being the most specific and direct way of estimating diaphragmatic strength, it is rarely used in clinical practice due to difficulty in doing the test.

Electrophysiological tests for diaphragm. The role of these tests in diaphragmatic paralysis is limited due to the many technical challenges of performing these tests, and complexity in their interpretation. They can assist in differentiating neuropathic and myopathic dysfunction [59,60]. 

## 8. Treatment

The treatment of diaphragmatic palsy depends on both the cause of it and the amount of impairment in respiratory functions. The concurrent medical conditions known to compromise respiratory functions like obesity and chronic obstructive pulmonary disease should be adequately treated before definitive treatment for diaphragmatic palsy. Inspiratory muscle training has shown to improve diaphragmatic pressure in patients with chronic obstructive pulmonary disease, spinal cord injury and post cardiac bypass diaphragmatic palsy [61,62,63,64,65,66,67]. 

Most patients with unilateral paralysis do not require any treatment. As most causes of hemi-diaphragm DP are transient, complete recovery is expected with time. Some patients may benefit from nocturnal ventilator support. If the weakness persists for more than a year, diaphragmatic plication can be done. In this procedure, the flaccid hemidiaphragm is made taut by over sewing the membranous central tendon and the muscular peripheral part. This will decrease the workload for the normal contralateral hemidiaphragm and improve ventilation/perfusion of the ipsilateral lung base. In recent times, video assisted thoracic surgery is used for diaphragmatic plication. In selected patients, this procedure has been shown to decrease dyspnea and improve functional capacity [68,69]. However, this procedure should not be done in patients with morbid obesity and progressive neuromuscular disease. Bilateral diaphragmatic paralysis should not undergo plication.

For bilateral diaphragmatic weakness, noninvasive positive pressure ventilation is the viable non-surgical treatment option. The criteria for starting this therapy is not very clear, but most experts recommend treatment if the partial pressure of carbon dioxide is more than 45 mm Hg or higher, and oxygen saturation is less than 88%. In patients with progressive neuromuscular disease leading to DP with reduction of maximum inspiratory pressure of less than 60 mm of mercury, reduction of forced vital capacity of less than 50% or absolute reduction of vital capacity of less than 20 mL per kg will benefit from noninvasive positive pressure ventilation [70,71,72,73,74]. With progression of the disease, tracheostomy with mechanical ventilation may be required. Early and prompt initiation of noninvasive pressure ventilation has been shown to improve lung functions, quality of life and overall survival [70,71,72,73,74]. Selected patients with bilateral diaphragmatic paralysis with persistent respiratory failure and intact phrenic nerve may benefit from phrenic nerve pacing [75,76,77,78,79]. In this procedure, the phrenic nerves are electrically stimulated using implanted electrodes to restore physiological functions of diaphragm. The electrodes can be inserted at the thoracic level, or on the abdominal surface of the diaphragm. Patients with high level cervical cord injury on machinal ventilation for at least three months showed significant improvement in their outcomes and some patients were completely liberated from the ventilator. This procedure is technically challenging and, despite improvement in the pacing technology, the diaphragmatic response is not sustained. Laparoscopic mapping of motor units and direct intramuscular simulation of diaphragmatic muscles have been tried with good results. 

## 9. Prognosis

The prognosis of patients with diaphragmatic paralysis is highly variable and depends on the cause of palsy. Most patients with unilateral diaphragmatic palsy will recover completely. Patients with DP after traumatic cause have a better prognosis than patients with idiopathic cause. Bilateral diaphragm weakness due to amyotrophy can show late improvement but patients with degenerative myopathy and neuropathy show a gradual progression to permanent respiratory failure. Patients with high cord cervical injury are also known for spontaneous recovery with supportive care. 

## 10. Conclusions

Diaphragmatic paralysis can be caused by many conditions and can lead to respiratory failure. Thoracic ultrasound has been increasingly used for evaluation of diaphragmatic functions. Most patients with unilateral DP are detected incidentally and do not require any specific management, while most patients with bilateral DP require permanent ventilator support. Selected patients with persistent respiratory failure can benefit from diaphragmatic plication or phrenic nerve pacing. 

## Supplementary Materials

The following are available online at http://www.mdpi.com/2079-9721/6/1/16/s1.

Supplementary File 1
