# Supplementary material for: Diaphragmatic Palsy
Source: Diseases. 2018 Feb 13;6(1):16. doi: 10.3390/diseases6010016 (PMC5871962; doi:10.3390/diseases6010016)
Supplement: Supplementary File 1 [file diseases-06-00016-s001.zip › New folder/legends of images and videos.docx]

Image 1. Chest x ray Pre and Post Cardiac Bypass Surgery(CABG) showing elevated left diaphragm after surgery. The sternal metal suture wires also seen.

Image 2. Chest x ray and Static Sniff test of patient with cervical spondylosis leading to phrenic nerve compression and left diaphragmatic palsy. Top panel showing chest x ray with left diaphragm A compared to normal chest x ray 2 years back. Lower panel. Static Sniff test shows no movement of diaphragm with sniffing

Image 3. Chest x ray PA and Lateral view showing right diaphragm located more than 2 intercostal spaces compared to left side.

Image 4. CT chest of patient with left diaphragmatic palsy showing abdominal contents beside heart and at higher level compared to liver suggesting left diaphragmatic palsy

Image 5. Spirometry of patient with bilateral diaphragmatic palsy showing significant drop of lung functions on supine position.

Video 1. Fluoroscopic Sniff testing of patient with left diaphragmatic palsy showing paradoxical movements of left hemidiaphragm.

Video 2. M mode Ultrasound of patient with right diaphragmatic palsy showing absence movements of right diaphragm with deep inspiration.
