# Supplementary figures and images for: Diaphragmatic Palsy
Source: Diseases. 2018 Feb 13;6(1):16. doi: 10.3390/diseases6010016 (PMC5871962; doi:10.3390/diseases6010016)

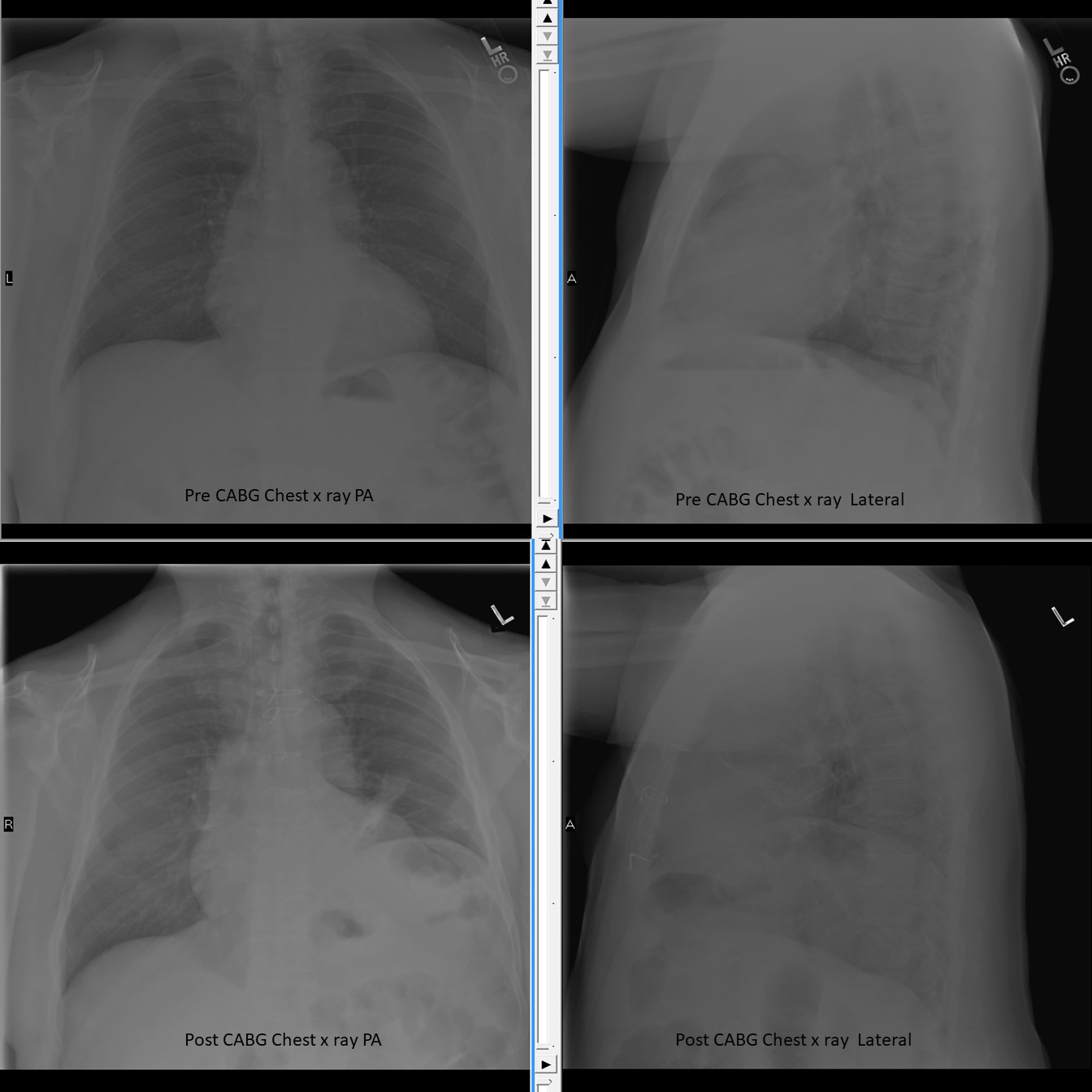

Supplement: Supplementary File 1 [file diseases-06-00016-s001.zip › New folder/Image 1.jpg]

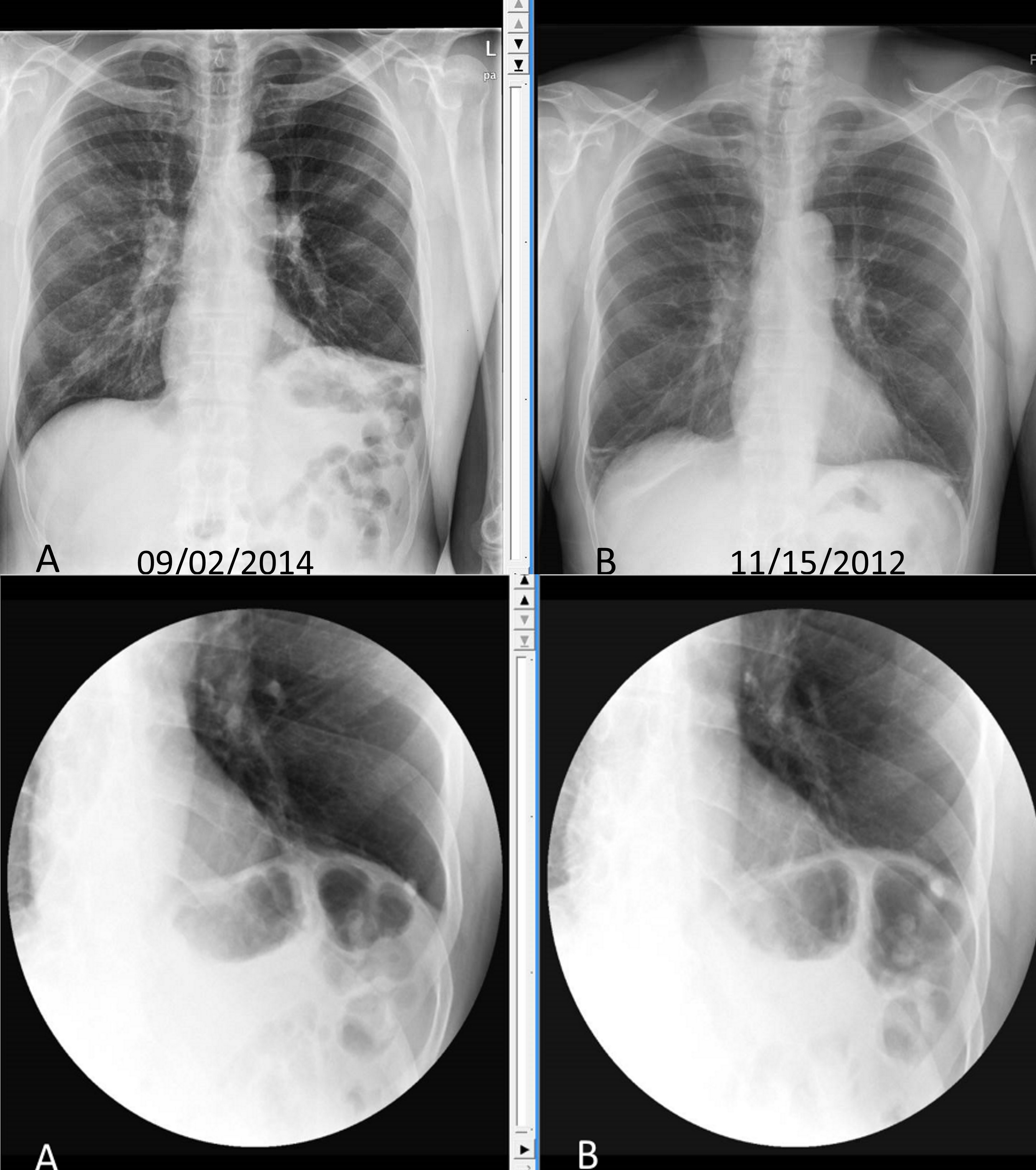

Supplement: Supplementary File 1 [file diseases-06-00016-s001.zip › New folder/Image 2.jpg]

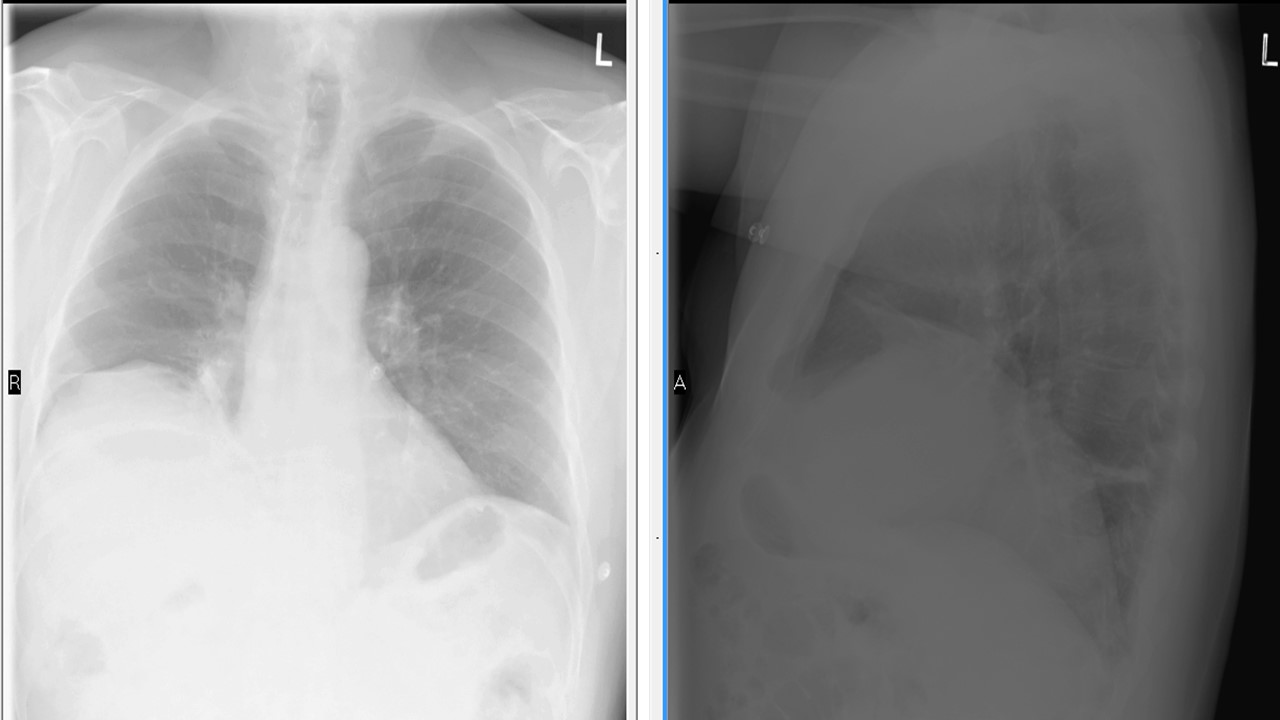

Supplement: Supplementary File 1 [file diseases-06-00016-s001.zip › New folder/Image 3..jpg]

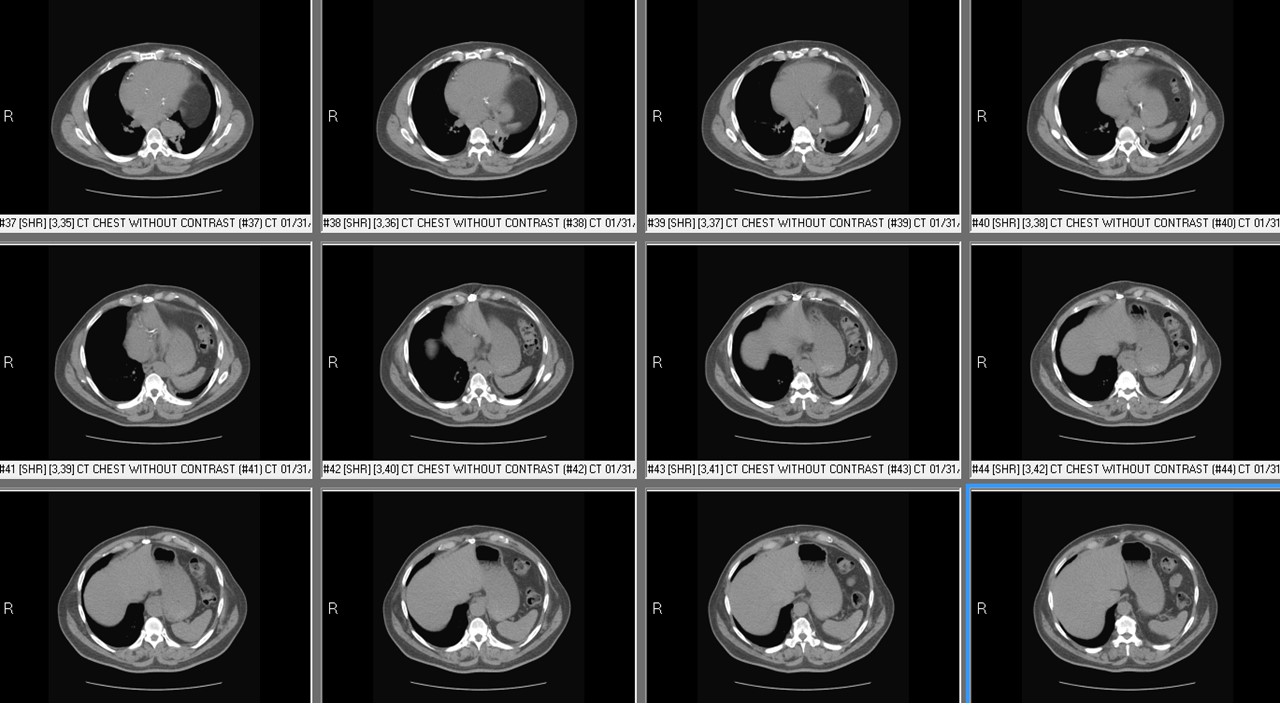

Supplement: Supplementary File 1 [file diseases-06-00016-s001.zip › New folder/Image 4.jpg]

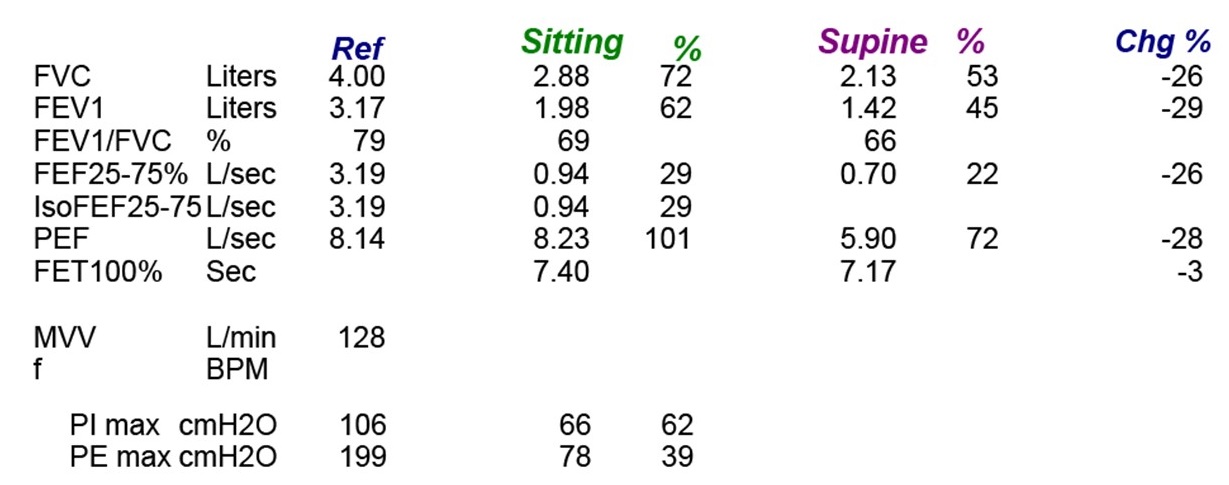

Supplement: Supplementary File 1 [file diseases-06-00016-s001.zip › New folder/Image 5.jpg]
